# Using nonlinear dynamics analysis to evaluate time response of cupping therapy with different intervention timings on reducing muscle fatigue

**DOI:** 10.3389/fbioe.2024.1436235

**Published:** 2024-10-01

**Authors:** Yuanyuan Jia, Yining Liu, Juntian Lei, Huihui Wang, Rong Wang, Pengrui Zhao, Tingting Sun, Xiao Hou

**Affiliations:** ^1^ Key Laboratory of Exercise and Physical Fitness, Beijing Sport University, Ministry of Education, Beijing, China; ^2^ School of Sport Science, Beijing Sport University, Beijing, China; ^3^ Harrison International Peace Hospital, Hengshui, China; ^4^ School of Sports Medicine and Rehabilitation, Beijing Sport University, Beijing, China

**Keywords:** cupping therapy, pre-condition, post-condition, nonlinear dynamics analysis, muscle fatigue

## Abstract

**Background:**

Cupping therapy has been indicated effective in reducing muscle fatigue after 24 h based on the spectral analyses of surface electromyography (sEMG). However, there is no sufficient evidence showing changes of sEMG nonlinear indexes at more time points after cupping therapy. Furthermore, it is unclear whether the intervention timings of cupping therapy affect the recovery from muscle fatigue. The purpose of this study was to use the sEMG nonlinear analysis to assess the difference of time response of cupping therapy between different intervention timings after muscle fatigue.

**Materials and methods:**

This randomized controlled trial recruited 26 healthy volunteers. Cupping therapy (−300 mmHg pressure for 5 min by the 45 mm-diameter cup) was applied before (i.e., pre-condition) or after (i.e., post-condition) muscle fatigue induced by performing repeated biceps curls at 75% of the 10 repetitions of maximum (RM) on the non-dominant upper extremity. Subjects were randomly allocated to the pre-condition group or the post-condition group. The sEMG signals during the maximal voluntary isometric contractions (MVC) of the biceps were recorded at four time points (i.e., baseline; post 1: immediate after cupping-fatigue/fatigue-cupping interventions; post 2: 3 h after cupping-fatigue/fatigue-cupping interventions; post 3: 6 h after cupping-fatigue/fatigue-cupping interventions). Two nonlinear sEMG indexes (sample entropy, SampEn; and percent determinism based on recurrence quantification analysis, %DET) were used to evaluate the recovery from exercise-introduced muscle fatigue. The Friedman test followed by the Nemenyi test and the Mann-Whitney U test were applied in statistics.

**Results:**

The SampEn and %DET change rate did not show any significant differences at four time points in the pre-condition group. However, there were significant delayed effects instead of immediate effects on improving muscle fatigue in the post-condition group (SampEn change rate: baseline 0.0000 ± 0.0000 vs. post 2 0.1105 ± 0.2253, *p* < 0.05; baseline 0.0000 ± 0.0000 vs. post 3 0.0627 ± 0.4665, *p* < 0.05; post 1–0.0321 ± 0.2668 vs. post 3 0.0627 ± 0.4665, *p* < 0.05; and %DET change rate: baseline 0.0000 ± 0.0000 vs. post 2–0.1240 ± 0.1357, *p* < 0.01; baseline 0.0000 ± 0.0000 vs. post 3 0.0704 ± 0.6495, *p* < 0.05; post 1 0.0700 ± 0.3819 vs. post 3 0.0704 ± 0.6495, *p* < 0.05). Moreover, the SampEn change rate of the post-condition group (0.1105 ± 0.2253) was significantly higher than that of the pre-condition group (0.0006 ± 0.0634, *p* < 0.05) at the post 2 time point. No more significant between-groups difference was found in this study.

**Conclusion:**

This is the first study demonstrating that both the pre-condition and post-condition of cupping therapy are useful for reducing muscle fatigue. The post-condition cupping therapy can e ffectively alleviate exercise-induced muscle fatigue and there is a significant delayed effect, especially 3 h after the interventions. Although the pre-condition cupping therapy can not significantly enhance muscle manifestations, it can recover muscles into a non-fatigued state.

## Introduction

Muscle fatigue, defined as the inability to generate a constant force after sustained or intense muscle contractions (Barry and Enoka, 2007), is traditionally categorized into central fatigue (central origin, the involved mechanism relates to the spinal and supra-spinal tract) and peripheral fatigue (peripheral origin, the involved mechanism relates to the structures distal of the neuromuscular junction) (Rampichini et al., 2020). Muscle fatigue can have detrimental effects. For athletes, muscle fatigue may impact physical and tactical performance (Coutinho et al., 2018), increasing risks of tissue injuries (i.e., tear, rupture, and fracture) (Edwards, 2018; Silva et al., 2018), which may lead to poor grades. For the general population, long-term muscle fatigue from the long-term stable motions in daily work results in a high susceptibility to musculoskeletal diseases (Punnett and Wegman, 2004). Consequently, identifying effective therapies for mitigating muscle fatigue is paramount.

Cupping therapy, a form of complementary and alternative medicine (CAM) utilizing negative pressure, has been employed to ameliorate exercise-induced muscle status. There are evidences that cupping therapy is useful for the increase of local blood flow (Hou et al., 2020), the reduction of muscle pain (Lauche et al., 2012), and the release of muscle stiffness (Jan et al., 2021). And all of these therapeutic effects may contribute to alleviate exercise-induced muscle fatigue. For example, one repeated-measures study investigated the effectiveness of a single cupping therapy compared to a sham-cupping therapy in twelve healthy untrained participants following a muscle fatigue protocol. Significant differences in surface electromyography (sEMG) linear indexes (mean frequency, MNF; median frequency, MDF; and spectral moments ratio, SMR) were described after 24 h rather than immediately after fatigue and cupping/sham-cupping interventions, which showed that cupping therapy reduced muscle fatigue significantly with a delayed effect (Hou et al., 2021). Based on these, further research is needed to delineate the conditions under which cupping therapy achieves optimal efficacy.

Intervention timings could influence the therapeutic effects of CAM. They can be divided into pre-condition (i.e., CAM therapies are applied before the exercise training) and post-condition (i.e., CAM therapies are applied after the exercise training). Different intervention timings of CAM therapy may yield distinct effects. For instance, Tuina is a Chinese therapeutic massage with CAM. Wei et al. has demonstrated that the Tuina after exercise (post-Tuina) was better to enhance the Ca^2+^ and Ca^2+^-adenosine triphosphatase (ATPase) concentration than the Tuina before exercise (pre-Tuina) 24 h after Tuina-exercise/exercise-Tuina interventions (Wei et al., 2022). It implied that post-Tuina could be the optimal strategy for promoting the skeletal muscle mitochondrial Ca^2+^-ATPase activity and the ability of mitochondria to transport Ca^2+^ to protect muscle tissue (Cully et al., 2017). Similarly, cupping therapy exerts its healing effects through mechanical shear strain on the soft tissue (Tham et al., 2006; Jan et al., 2021), which is analogous to the mechanical shear stress applied during massage (Smith et al., 1994). Therefore, significant differences may exist between pre-condition and post-condition cupping therapy. However, evidence regarding the effects of different intervention timings on cupping therapy remains insufficient. Moreover, current evidence indicates that the delayed effects of post-condition cupping therapy only manifested 24 h after muscle fatigue (Hou et al., 2021; Liao et al., 2021). Little is known about the more detailed time response for pre-condition and post-condition cupping therapy. Therefore, it is necessary to delve into additional time points for both pre-condition and post-condition to elucidate the time response of cupping therapy in alleviating muscle fatigue.

The sEMG can record the continuous myoelectric activity by the non-invasive electrodes placed on the local skin (González-Izal et al., 2012). It has been used to detect the effects of cupping therapy after exercise-induced muscle fatigue. The researchers validated the recovery effects of cupping therapy on fatigued muscles through sEMG linear analyses (such as MDF, MNF, SMR) (Chen et al., 2018; Hou et al., 2021). However, the aforementioned analytical methods originated from traditional linear dynamics, which were the most common analytical tools for describing two-body problems (linear systems) before the maturity of nonlinear dynamics theory. On the other hand, researchers have demonstrated that sEMG signals exhibited with nonlinear chaotic characteristics (Erfanian et al., 1996; Meng and Liu, 2006), which was conducted by the complex nonlinear neuromuscular system (Röhrle et al., 2019). So the nonlinear analysis (e.g., entropy, recurrence quantification analysis, and so on) seems better to assess information on fatigue-induced changes of neuromuscular processes that could be ignored by the linear analysis approaches (Merletti and Parker, 2004). Subsequent research should employ nonlinear analyses to elucidate the underlying mechanisms of sEMG signal changes following cupping therapy.

In a word, it remains unclear whether the intervention timings of cupping therapy affect the recovery from muscle fatigue. And there is insufficient evidence demonstrating changes in sEMG nonlinear indices at multiple time points after cupping therapy. This study aims to employ the sEMG nonlinear methods to detect the difference in time response of cupping therapy between various intervention timings following muscle fatigue. Specifically, our hypothesis is that post-condition cupping therapy yields superior results compared to pre-condition cupping therapy, with a delayed effect on reducing exercise-induced muscle fatigue.

## Methods

### Study design

A parallel-group randomized controlled trial (RCT) was conducted in this study. According to different intervention timings, participants were randomly assigned to a group of either: 1) pre-condition group: cupping therapy conducted before biceps brachii fatigue protocol (i.e., cupping-fatigue), or 2) post-condition group: cupping therapy conducted after biceps brachii fatigue protocol (i.e., fatigue-cupping). Assessments were conducted at baseline (before cupping therapy and fatigue protocol), post 1 (immediately after cupping-fatigue/fatigue-cupping interventions), post 2 (3 h after cupping-fatigue/fatigue-cupping interventions) and post 3 (6 h after cupping-fatigue/fatigue-cupping interventions) by sEMG during the maximum voluntary contraction (MVC) of biceps brachii.

### Participants

The recruitment process for this study was conducted at Beijing Sport University (BSU) through advertisements and word-of-mouth. Prospective participants underwent a rigorous prescreening procedure via an electronic questionnaire to ensure adherence to specific eligibility criteria (shown in Supplementary Appendix SA). Ethical approval for the study was obtained from the Sports Science Experiment Ethics Committee of BSU (Approval No. 2023022H), and informed consent was obtained from all participants. The sample size was calculated as 24 based on the power analysis with an assumption of a very large effect size (1.2), alpha level at 0.05, power at 0.95 and two groups for the independent samples t-test. This effect size choice was informed by previous literature demonstrating the impact of cupping therapy on sEMG linear indexes (Hou et al., 2021). Considering the drop-out, the sample size was determined as 26, 13 per group.

Participants were evenly allocated to two groups using a random number table method. We used Excel to generate the respective random numbers for each participant. Subsequently, participants were sorted in descending order based on their random numbers, with the top half allocated to the pre-condition group and the rest allocated to the post-condition group.

### Cupping therapy

**FIGURE 1:**
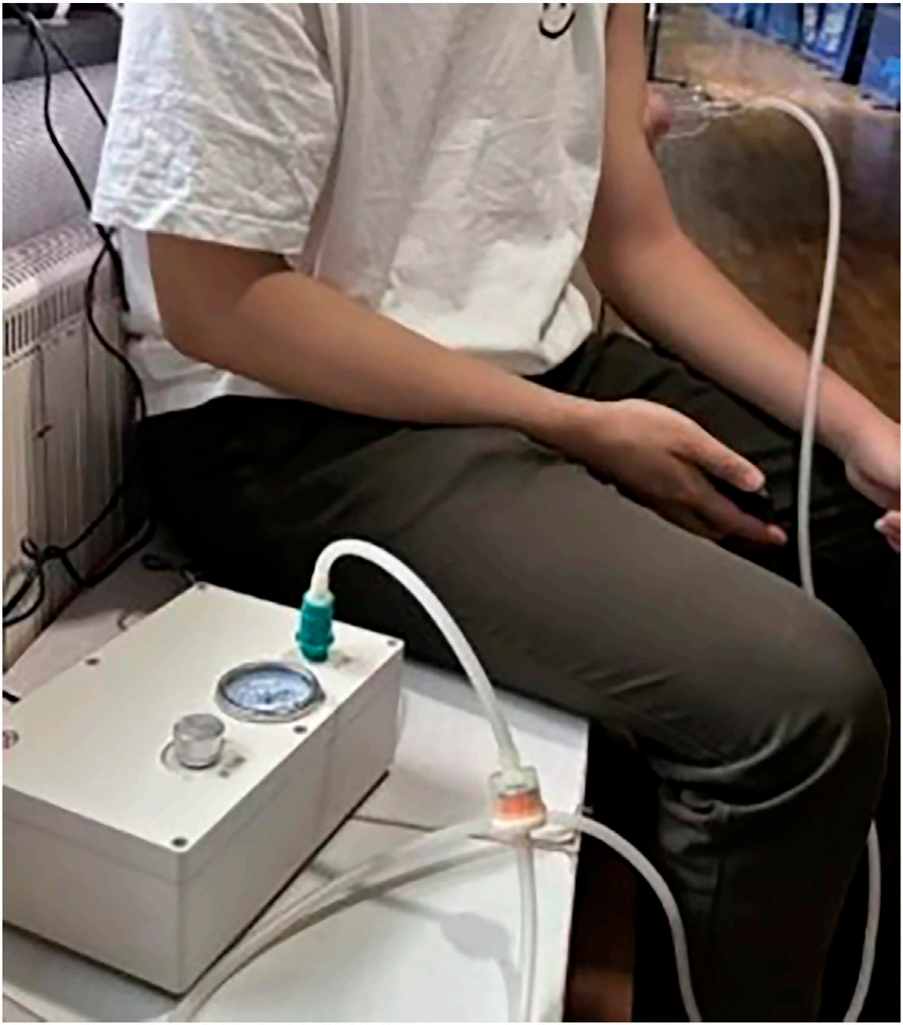
The photograph of the cupping device.

**FIGURE 2:**
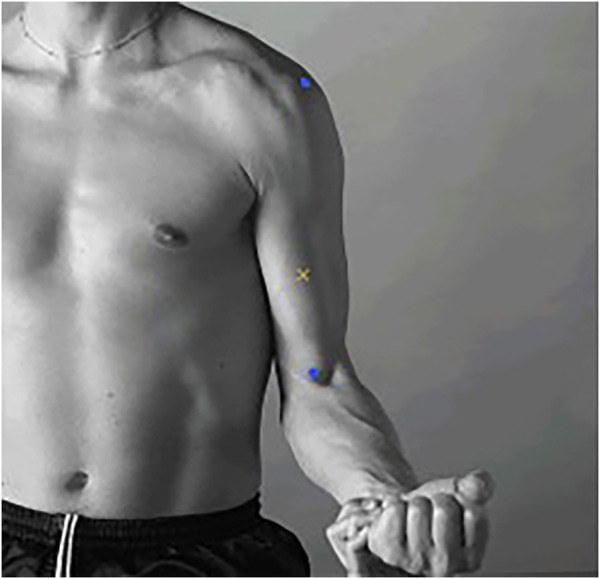
The abdominal center of the biceps brachii. (Notes: The two blue markers represent the medial acromion and the fossa cubit from top to bottom. The yellow cross represents the abdominal center of the biceps brachii).

Cupping therapy in this study utilized a cup of 45 mm inner diameter with the negative pressure of 300 mmHg for 5 min conducted by an electric negative pressure gauge (MF-H96, Baoyizhen, China; see in Figure 1). According to a previous study, the cupping therapy dose has been indicated the significant improvement on muscle fatigue. The treatment site was at the abdominal center of the biceps brachii (on the line between the medial acromion and the fossa cubit at 1/3 of the way from the fossa cubit; see the yellow cross in Figure 2) (Hou et al., 2021).

### sEMG assessment

**FIGURE 3:**
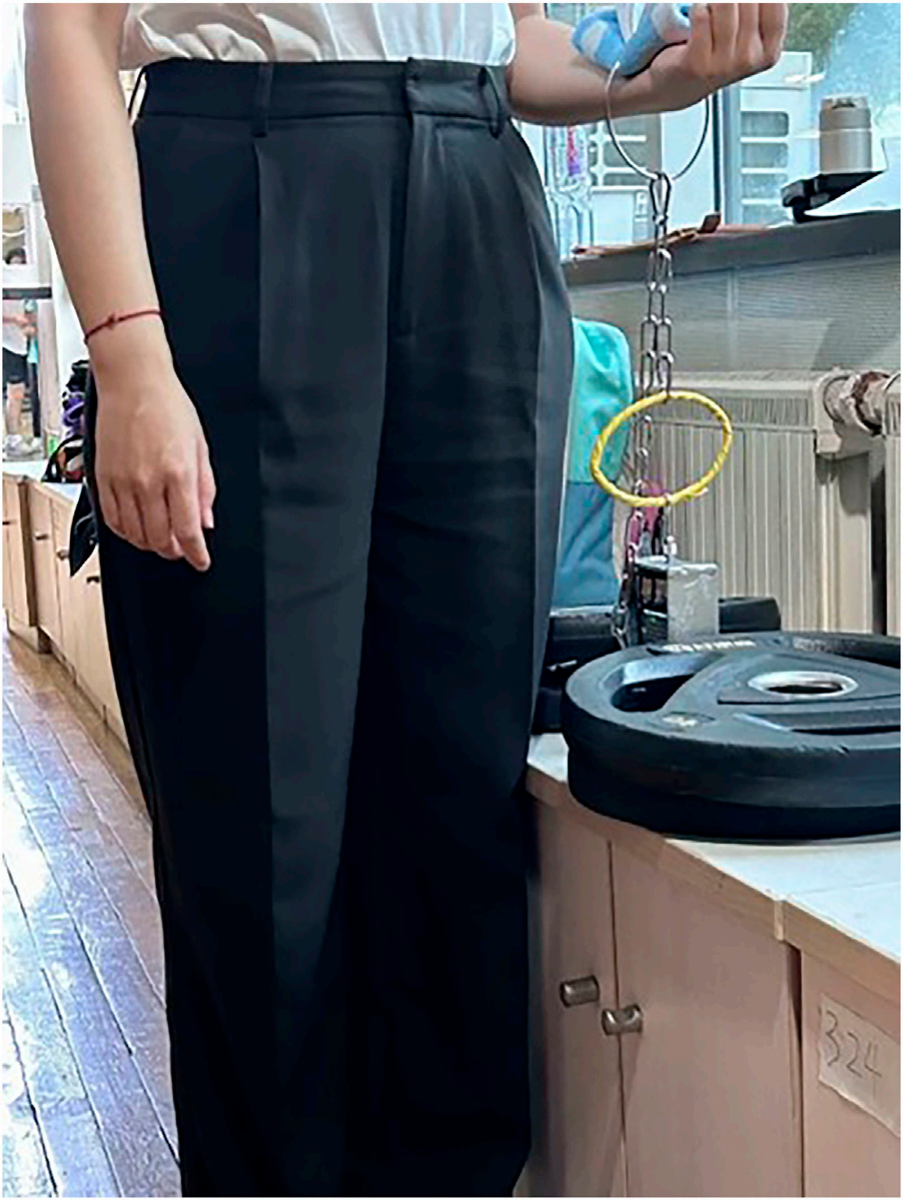
The photographs of the MVC test.

The sEMG assessment comprised three 5-s MVC tests of the biceps brachii, each separated by a 60-s interval. Signals were recorded using a wireless EMG system (Delsys, Inc., Natick, MA) with a 16-channel sensor setup, sampled at 1,000 Hz. Sensors were affixed with 3M tape and muscle patches for stability. Participants performed the MVC test by exerting maximal force on a force transducer (DY920, Freud, Daysensor, China) while maintaining a 90° angle at the elbow of their non-dominant arm (Hermens et al., 2000). The force transducer, inverted onto the platform and secured with a weight, was pulled perpendicular to it (see in Figure 3). Participants monitored sEMG tracings on a computer screen to stabilize force output. Sensor placement was standardized at the abdominal center of the biceps brachii, and an indelible marker was used to ensure consistent positioning for each intervention (Hermens et al., 2000).

### Experimental procedures

**FIGURE 4:**
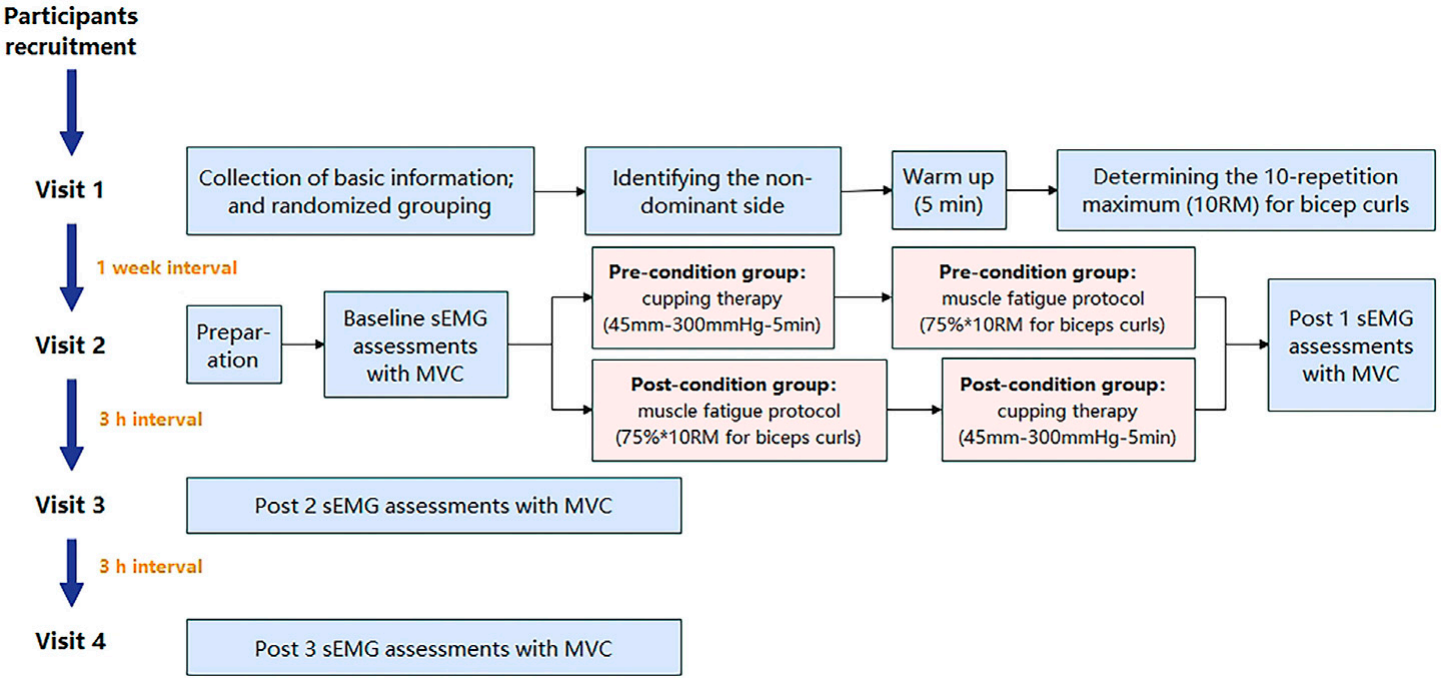
The flowchart of experimental procedures. (Note: sEMG: surface electromyography; MVC, maximum voluntary contraction).

The flowchart in Figure 4 shows the experimental procedures. Participants in both groups (pre-condition and post-condition) completed 4 visits to assess basic information (Visit 1), immediate effects (Visits 2) and delayed effects (Visits 3 and 4). Before the experiment, participants relaxed in a seated position for ≥ 5 min during each visit.

During the Visit 1, an online questionnaire was completed for basic information and inclusion/exclusion criteria. The Edinburgh Handedness Inventory was then administered to determine the non-dominant arm (Veale, 2014), minimizing daily-life-induced muscle fatigue. After signing of the “Participant Information and Informed Consent Form,” participants engaged in a 5-min warm-up involving unloaded dynamic bicep curls on the non-dominant side. Subsequent tests determined the one-repetition maximum (1RM) for bicep curls using varying dumbbell weights, with a 3-min rest period between attempts. A formula was utilized to calculate the 10-repetition maximum (10 RM): $1RM=8.841+\left(1.1828\times 7-10RM\right)$ (Abadie et al., 1999), with 75% of this weight applied for the second test to induce muscle fatigue.

During the Visit 2, participants were calibrated to the test position. Baseline sEMG assessments were conducted after skin preparation with 70% isopropyl alcohol. After a 3-min rest, participants underwent the first sEMG assessment. Referring to previous studies, the muscle fatigue protocol was isolated biceps curls with 75% of 10 repetition maximum (RM) until exhaustion of the non-dominant arm at the pace of 15 repetitions per min (Hou et al., 2021). The pre-condition group received “cupping-fatigue” interventions (cupping: cupping therapy; and fatigue: muscle fatigue protocol), while the post-condition group received “fatigue-cupping” interventions. Immediate post 1 sEMG assessments were conducted.

At 3 h (post 3) and 6 h (post 4) after Visit 2, participants performed three 5-s MVC tests with sEMG assessment twice (Visit 3 and 4), following the detailed process described above.

### EMG analysis

The EMG signals were filtered of a zero-lag band pass filter and processed by absolute value using the EMGworks Analysis (Delsys, Inc., Natick, MA). Then, two nonlinear dynamic analysis methods were applied to evaluate the effect of cupping therapy on alleviating muscle fatigue using Python. Sample entropy (SampEn) quantifies time series entropy by assessing the likelihood that similar sequences remain similar within epochs (Richman and Moorman, 2000). It has been applied to detect muscle fatigue. The SampEn in this study was calculated as the following formula (Eq. 1):

$$SampEn\left(m,r,N\right)=-\log\left(\frac{\sum_{i=1}^{N\;-m}A_{i}}{\sum_{i=1}^{N\;-m}B_{i}}\right)=-\log\left(\frac{A}{B}\right)$$ (1)

where *N* is the number of data points in the time series (a resample frequency of 250 Hz and a segment length of 5 s), *m* is the length of the template (taken as 2), *r* is the similar capacity of each signal segment, *A*_*i*_ is taken to be 1 in the similar case and 0 in the opposite case, *A* is the total number of matches of the *i*th template of length *m* + 1 data points, *B*_*i*_ and *B* are the number of matches of the *i*th template of length *m* data points (Richman and Moorman, 2000).

Another nonlinear dynamic method, the recurrence quantification analysis (RQA) identifies recurring patterns and non-stationarities (Caballero-Pintado et al., 2018). Percent determinism (%DET), a common RQA measure, calculates the ratio of connected diagonals to all ones in the matrix (Webber Jr and Zbilut, 2007). The %DET in this study was calculated as the following formula (Eq. 2):

$$\%\mathrm{DET}=\frac{\sum_{l=l_{\min}}^{N}lP\left(l\right)}{\sum_{i,j=1}^{N}R_{i,j}}$$ (2)

where *l* is the length of the line segment, *l*_*min*_ is the minimum length of the line segment (generally taken as 2), *P(l)* is the number of line segments parallel to the main diagonal and of length *l*, and the *R*_*i*,__*j*_ represents the points on the recurrence plot.

The SampEn and the %DET were computed from the most forceful of three 5-s MVC tests. The SampEn and the %DET change rates were then utilized to evaluate cupping therapy’s impact on fatigue reduction. These rates represent the difference between SampEn or %DET at various time points and their respective baseline values, divided by the baseline values. For instance, considering SampEn, the formula was as follows (Eq. 3):

$$SampEn\;chage\;rate=\frac{{SampEn}_{\;at\;a\;given\;time}-{SampEn}_{\;baseline}}{{SampEn}_{\;baseline}}$$ (3)

### Statistical analysis

All statistical analyses were performed by the SPSS (Version 26, Chicago, IL, United States). Firstly, the normality of the results was checked using Shapiro–Wilk tests. The Friedman test, with Nemenyi post-hoc test was performed to examine the differences in the SampEn and the %DET change rate among different time points within both the pre-condition and the post-condition groups. The differences in the SampEn and the %DET change rate between pre-condition cupping therapy and post-condition cupping therapy at various time points were examined using Mann-Whitney U tests.

## Results

In this study, 26 healthy, young participants (8 male and 18 female) were recruited for this study. No participants dropped out during the trail. The demographic data and basic characteristics were as follows: age (years) = 20.35 ± 1.32; height (m) = 1.69 ± 0.08; weight (kg) = 65.28 ± 13.76; body mass index (BMI, kg/m^2^) = 22.74 ± 4.32; and the maximum force value output during MVC (Newton): baseline-value = 195.64 ± 93.76, post 1-value = 169.51 ± 90.62, post 2-value = 176.17 ± 93.30, post 3-value = 171.33 ± 106.76. Only two of the 26 participants were left-handed. The specifics of the pre-condition and post-condition cupping therapy group were presented in Table 1.

**TABLE 1:** Demographic data and basic characteristics.

	Pre-condition group (N = 13)	Post-condition group (N = 13)
Age, mean (SD), yr	20.08 (1.44)	20.62 (1.19)
Female (%)	69.23%	69.23%
Height, mean (SD), m	1.69 (0.08)	1.69 (0.09)
Weight, mean (SD), kg	62.85 (12.39)	67.72 (15.11)
BMI, mean (SD), kg/m^2^	21.88 (2.81)	23.59 (5.43)
Right-handed (%)	84.62%	100.00%
Maximum force value output during MVC, Newton		
Baseline	206.85 (109.96)	184.43 (77.16)
Post 1	179.32 (105.02)	159.71 (76.63)^a^
Post 2	183.13 (105.18)	169.21 (83.47)
Post 3	176.67 (115.46)^a^	165.98 (101.75)^a^

Note: BMI, body mass index; MVC, maximum voluntary contraction. The symbols ^a^ indicates *p* < 0.05 compared to baseline.

### The SampEn change rate

**FIGURE 5:**
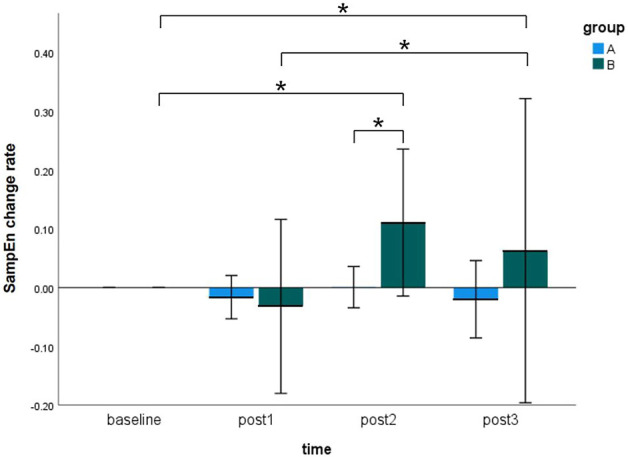
Comparison of the SampEn change rate within-groups and between-groups at all time points. (Notes: A: the pre-condition group; B: the post-condition group; post1: immediate after interventions; post 2: 3 h after interventions; post 3: 6 h after interventions. The symbols * indicates *p* < 0.05).

Figure 5 showed the SampEn change rate of the sEMG signals during MVC tests at four time points (i.e., baseline, post 1, post 2, and post 3) in the pre-condition cupping therapy group and the post-condition cupping therapy group.

In case of the pre-condition group, the SampEn change rate did not show any significant differences at four time points. In case of the post-condition group, the SampEn change rate showed significant increases from baseline to post 2 (baseline 0.0000 ± 0.0000 vs. post 2 0.1105 ± 0.2253, *p* < 0.05), from baseline to post 3 (baseline 0.0000 ± 0.0000 vs. post 3 0.0627 ± 0.4665, *p* < 0.05), and from post 1 to post 3 (post 1–0.0321 ± 0.2668 vs. post 3 0.0627 ± 0.4665, *p* < 0.05). It meant that delayed effects of cupping therapy were found in the post-condition group rather than immediate effects. Moreover, the SampEn change rate of the post-condition group (0.1105 ± 0.2253) was significantly higher than that of the pre-condition group (0.0006 ± 0.0634, *p* < 0.05) at the post 2 time point. No more significant between-groups difference was found in this study. The specifics of two groups were presented in Supplementary Appendix SB. The original results of SampEn are included in Supplementary Appendix SC.

### The %DET change rate

**FIGURE 6:**
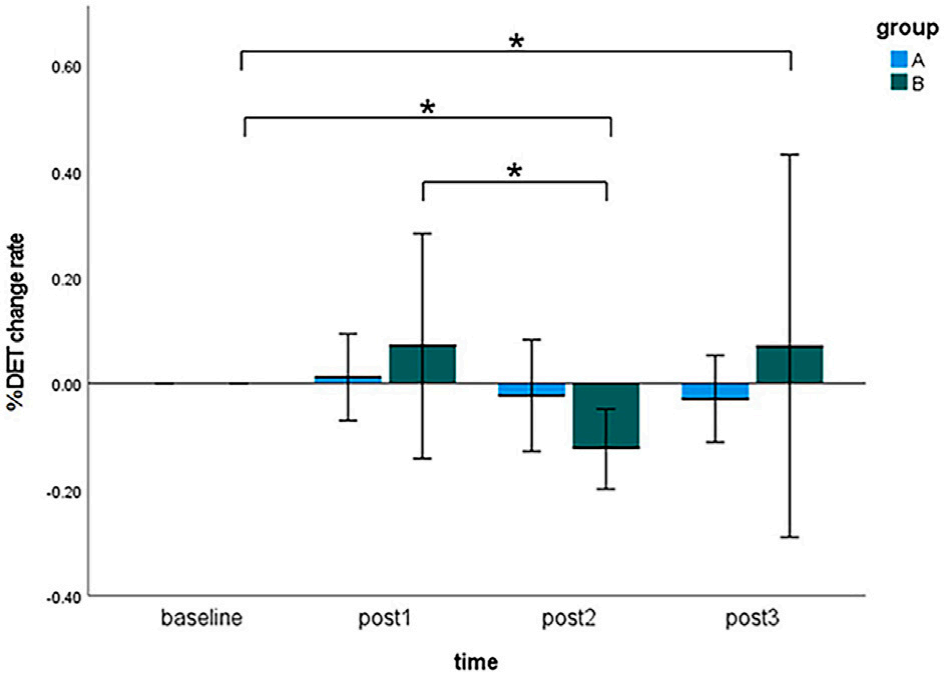
Comparison of the %DET change rate within-groups and between-groups at all time points. (Notes: A: the pre-condition group; B: the post-condition group; post1: immediate after interventions; post 2: 3 h after interventions; post 3: 6 h after interventions. The symbols * indicates *p* < 0.05).

Figure 6 visualized the results of the %DET change rate of the sEMG signals during MVC tests at four time points (i.e., baseline, post 1, post 2, and post 3) in the pre-condition cupping therapy group and the post-condition cupping therapy group.

When cupping therapy was performed before muscle fatigue (pre-condition), there was no significant differences of the %DET change rate at all time points. When cupping therapy was performed after muscle fatigue (post-condition), there were significant delayed effects instead of immediate effects on improving muscle fatigue in the post-condition group (baseline 0.0000 ± 0.0000 vs. post 2 −0.1240 ± 0.1357, *p* < 0.01; baseline 0.0000 ± 0.0000 vs. post 3 0.0704 ± 0.6495, *p* < 0.05; post 1 0.0700 ± 0.3819 vs. post 3 0.0704 ± 0.6495, *p* < 0.05). When comparing the %DET change rate between the pre-condition group and the post-condition group, the differences were not significant at all time points. The specifics of two groups were presented in Supplementary Appendix SB. The original results of %DET are included in Supplementary Appendix SC.

## Discussion

The study indicated the efficacy of both the pre-condition and the post-condition cupping therapy in mitigating muscle fatigue. Notably, significant delayed effects were observed in the post-condition group instead of immediate effects. The pre-condition cupping therapy demonstrated the ability to restore muscles to a non-fatigued state. Particularly, results indicated superiority of the post-condition group over the pre-condition group, evidenced by SampEn at post 2 time points, which corroborated our initial hypothesis. To the best of our knowledge, this study is the first to detect differences in the time response of cupping therapy between different intervention timings after muscle fatigue.

In this study, the methodological selection of nonlinear analysis was motivated by the following considerations. Nonlinear indices were preferred over traditional linear indices due to the limitations of linear analysis in capturing the complexity of the neuromuscular system. Linear methods attempt to isolate stable components but often oversimplify interactions among system elements. Nonlinear analysis methods provide a more comprehensive approach, hence their exclusive use in this paper. Among them, entropy analysis serves as an effective tool for assessing system complexity, describing regularity and predictability in time series data. Lower entropy values indicate more regular systems, reflecting reduced complexity (Rampichini et al., 2020). In the neuromuscular system, muscle fatigue is regarded as an unhealthy pathological state with decreased signal complexity, evidenced by declining entropy values (Şaylı and Çotuk, 2015). As a form of entropy analysis, SampEn shares these characteristics. Pethick et al. found a decrease in SampEn with increasing muscle fatigue during 100% MVC tasks (Pethick et al., 2015). Considering the physiological significance of SampEn, our study utilized it to evaluate the efficacy of cupping therapy in alleviating muscle fatigue. Results revealed no significant reduction in SampEn change rates in either the pre-condition or the post-condition cupping therapy groups compared to their respective baseline values. It suggested that cupping therapy with different intervention timings effectively prevented declines in neuromuscular system complexity following muscle fatigue. This aligns with findings by Liao et al. (2021), who observed significantly smaller reductions in entropy values among cupping group participants compared to sham cupping group participants following muscle fatigue.

Another sEMG nonlinear analysis method, %DET was also employed in this study. Consistent with SampEn findings, both the pre-condition and the post-condition cupping therapy effectively modulated muscle performance decline following exercise-induced muscle fatigue. Specifically, %DET monitors activity at the micro-level of the neuromuscular system (i.e., motor unit behavior). Both types of changes in motor units following fatigue can lead to alterations in %DET. Specifically, an increase in motor unit synchronization (MUS) and a decrease in motor unit conduction velocity (MUCV) result in an elevated %DET (Şaylı and Çotuk, 2015). And the parameters MUS and MUCV are respectively associated with central and peripheral fatigue respectively, resulting in %DET reflecting both central and peripheral fatigue. For example, a previous research indicated that high-intensity isometric contractions led to increased MUS, which was considered as an effective strategy by the central nervous system to counteract cortical fatigue (Kleine et al., 2001). On the other hand, peripheral fatigue involved the decrease in MUCV due to changes in electrochemical factors such as phosphate accumulation and intracellular pH increase (Brody et al., 1991). In this study, %DET results indicated no significant increases in the pre-condition group at all post-intervention time points (post 1, post 2, post 3), and in the post-condition group at the first two post-intervention time points (post 1, post 2), compared to their respective baseline values. It showed that cupping therapy effectively prevents post-fatigue increases in MUS and decreases in MUCV at these time points. Conversely, a significant increase in %DET at the post 3 compared to baseline indicated an elevation in MUCV and a decrease in MUS, reflecting a deepening level of fatigue.

Our results showed differences between pre-condition and post-condition. At the post 2 time point, the SampEn change rate in the post-condition cupping therapy group significantly exceeded that of the pre-condition group. This meant that post-condition cupping therapy could enhance neuromuscular system complexity 3 h after fatigue. Furthermore, the post-condition cupping therapy exhibited significant delayed improvement effects on fatigue-induced SampEn increase and %DET decrease, even surpassing baseline levels (i.e., a non-fatigued state). In contrast, pre-condition cupping therapy only restored the aforementioned muscle fatigue-related characteristics to a non-fatigued state.

For the differences between pre-condition and post-condition, the underlying mechanism might be attributed to the hemodynamics. The blood circulation promotion theory suggested a potential physiological mechanism by which cupping therapy improves muscle condition, involving local hemodynamic changes regulated by positive pressure under the cup rims and negative pressure under the cup during cupping. For the effects of positive pressure, studies indicated that 200 mmHg intermittent pressure caused by a cuff could induce intermittent vascular occlusion and promote blood flow reperfusion post-cuff removal, which was aided in muscle stiffness and fatigue (Nakajima et al., 2022). Moreover, during cupping therapy, compression of tissues at the cup rim generates pressure approximately an order of magnitude greater than the negative pressure under the cup (Tham et al., 2006). For instance, assuming a negative pressure of 300 mmHg under the cup, the corresponding positive pressure at the cup rim is approximately 3,000 mmHg. Compared to the 200 mmHg intermittent pressure, maintaining this pressure intensity for 5 min during cupping is sufficient to induce local blood flow reperfusion. In addition, tensile stress of tissue under the cup increases shear stress between blood flow in capillaries and endothelial cells, which stimulates endothelial cells to release more nitric oxide (NO) and promotes vasodilation (Moncada, 1992). Collectively, these mechanisms lead to enhanced local blood flow and accelerated circulation following cupping therapy. And the negative pressure suction also causes capillary rupture, leading to the extravasation of red blood cells into tissue fluid, manifested as petechiae and ecchymosis on the skin (Kim and Lee, 2014; Lowe, 2017). On the other hand, exercise training accompanies increased blood flow and changes in hemodynamics (Clifford, 2007). However, the intramuscular pressure generated during muscle contraction ranges from 270 to 570 mmHg (Sejersted et al., 1984), which is lower than the high-intensity compression and suction exerted externally. Thus, when cupping therapy and skeletal muscle contraction jointly regulate hemodynamics, the intervention sequence may be a critical factor influencing efficacy. In the pre-condition group, the reperfusion and NO effects after cupping therapy resulted in rapid increases in muscle blood flow and capillary rupture. Subsequent muscle contractions exacerbated local blood flow, further perfusing already damaged capillaries and aggravating skeletal muscle damage (de Carvalho et al., 2023). The cupping therapy before exercise training accelerated the clearance of metabolic waste and transport of nutrients under non-fatigued conditions. So it enhanced tissue status before exercise and facilitated post-fatigue recovery to a non-fatigued state. However, the pre-condition cupping therapy failed to effectively clear metabolic waste caused by subsequent muscle contractions, ultimately leading to limited muscle fatigue recovery. In the post-condition group, cupping therapy led to increased blood flow and accelerated blood circulation after exercise-induced muscle fatigue. It was most conducive to clearing metabolic waste, transporting nutrients and facilitating post-exercise muscle fatigue recovery. Based on these results, we suggested that post-condition cupping therapy had a greater advantage in improving exercise-induced muscle fatigue.

In addition to the different intervention timings, our study also indicated that post-condition cupping therapy exhibited significant delayed effects. The 3 h after intervention may be a critical time point for the effectiveness of cupping therapy on recovery muscle fatigue. One previous study has confirmed the delayed effect of the post-condition cupping therapy. Hou et al. (2021) found that cupping therapy effectively alleviated biceps brachii muscle fatigue at 24 h post-interventions based on sEMG linear indicators (i.e., MDF, MNF, and SMR). This seemingly contradicted our findings that cupping therapy significantly improved muscle fatigue at 3 h post-interventions. Although both studies employing sEMG as a measurement tool, their specific analytical methods and physiological implications differ, potentially contributing to inconsistent conclusions. Additionally, exercise-induced fatigue can be divided into peripheral and central fatigue. Peripheral fatigue phenomena involve changes in metabolic and structural components at the muscle level. Central nervous system regulates the peripheral system, and central fatigue affects peripheral fatigue. Hou et al. (2021) attributed changes in sEMG linear indicators to the effects of cupping therapy on peripheral fatigue phenomena such as post-exercise hydrogen ion (H^+^) accumulation and immune response. In our study, we employed nonlinear indicators to explore the modulation mechanism of cupping therapy on “central-peripheral” fatigue. Previous studies have shown that SampEn and %DET are associated with β-band coherence activity between the brain and muscles, reflecting cortico-muscular coherence (i.e., the central nervous system’s regulation of the peripheral system) (McManus et al., 2016). With the progression of muscle fatigue, the central nervous system’s regulatory capacity over the peripheral system decreases, as evidenced by reduced β-band cortico-muscular coherence (Yang et al., 2009). Our study showed that the post-condition cupping therapy significantly increased SampEn and decreased %DET at 3 h post-interventions compared to baseline. It suggested that the post-condition cupping therapy might enhance the β-band coherence activity beyond baseline levels. Considering the physiological significance of SampEn and %DET, the increased complexity of the neuromuscular system, decreased MUS, and increased MUCV after the post-condition cupping therapy might suggest with the improvement in β-band cortico-muscular coherence. Additionally, this regulation pathway involves fast neural transmission pathways so that the post-condition cupping therapy showed optimal therapeutic effects on the central fatigue level appearing at an earlier time.

To the best of our knowledge, this is the first study to detect the difference of time response of cupping therapy between different intervention timings after muscle fatigue based on the sEMG nonlinear dynamic analysis. However, this study has several limitations: 1) The study only collected sEMG signals at four time points: baseline, immediate, 3 h and 6 h after “cupping-fatigue” or “fatigue-cupping” tasks, without exploring additional time points. Further investigation into the time response of cupping therapy requires monitoring more time points in the future; 2) Different sEMG signal nonlinear analysis methods may reveal different physiological meanings of the neuromuscular system. This study only investigated the effects of cupping therapy on muscle fatigue improvement using two analysis methods (SampEn and %DET). Future research could explore the potential mechanisms of cupping therapy in restoring exercise-induced muscle fatigue using other algorithms.

## Conclution

This study is the first to demonstrate that both pre-condition and post-condition cupping therapy effectively reduce muscle fatigue. Cupping therapy conducted after muscle fatigue can effectively alleviate exercise-induced muscle fatigue and there is a significant delayed effect, especially 3 h after the intervention. Although conducting cupping therapy before exercise-induced muscle fatigue can not significantly enhance muscle manifestations, it can recover muscles into a non-fatigued state.

## Data Availability

The original contributions presented in the study are included in the article/Supplementary Material, further inquiries can be directed to the corresponding authors.
