# Supplementary material for: Using nonlinear dynamics analysis to evaluate time response of cupping therapy with different intervention timings on reducing muscle fatigue
Source: Front Bioeng Biotechnol. 2024 Oct 1;12:1436235. doi: 10.3389/fbioe.2024.1436235 (PMC11473309; doi:10.3389/fbioe.2024.1436235)
Supplement: Supplementary file 1 [file Table1.docx]

**Appendix B**

**Table** Comparison of the SampEn change rate [Mean (SD)] and the %DET change rate [Mean (SD)] between the pre-condition group and the post-condition group at all time points.

| **Variables** |  | **Baseline** | | | |  | **Post1 (0h)** | | | |  | **Post2 (3h)** | | | |  | **Post3 (6h)** | | | |
| --- | --- | --- | --- | --- | --- | --- | --- | --- | --- | --- | --- | --- | --- | --- | --- | --- | --- | --- | --- | --- |
|  |  | PRE | POST | Z | *P* |  | PRE | POST | Z | *P* |  | PRE | POST | Z | *P* |  | PRE | POST | Z | *P* |
| SampEn Change Rate |  | 0.0000 (0.0000) | 0.0000 (0.0000) | 0.000 | 1.000 |  | -0.0164 (0.0665) | -0.0321 (0.2668) | -0.282 | 0.778 |  | 0.0006 (0.0634) | 0.1105 (0.2253) | -2.179 | 0.029 |  | -0.0200 (0.1186) | 0.0627 (0.4665) | -1.615 | 0.106 |
| %DET  Change  Rate |  | 0.0000 (0.0000) | 0.0000 (0.0000) | 0.000 | 1.000 |  | 0.0114 (0.1476) | 0.0700 (0.3819) | -0.385 | 0.701 |  | -0.0232 (0.1896) | -0.1240 (0.1357) | -1.462 | 0.144 |  | -0.0292 (0.1477) | 0.0704 (0.6495) | -0.590 | 0.555 |

Notes: PRE: the pre-condition group; POST: the post-condition group.
