# Supplementary material for: Using nonlinear dynamics analysis to evaluate time response of cupping therapy with different intervention timings on reducing muscle fatigue
Source: Front Bioeng Biotechnol. 2024 Oct 1;12:1436235. doi: 10.3389/fbioe.2024.1436235 (PMC11473309; doi:10.3389/fbioe.2024.1436235)
Supplement: Supplementary file 2 [file Table2.docx]

**Appendix C**

Table Results of the SampEn [Mean (SD)] and the %DET [Mean (SD)] in the pre-condition group and the post-condition group at all time points.

| **Variables** |  | **Baseline** | |  | **Post 1 (0h)** | |  | **Post 2 (3h)** | |  | **Post 3 (6h)** | |
| --- | --- | --- | --- | --- | --- | --- | --- | --- | --- | --- | --- | --- |
|  |  | PRE | POST |  | PRE | POST |  | PRE | POST |  | PRE | POST |
| SampEn |  | 1.6710 (0.1075) | 1.5987 (0.2902) |  | 1.6395 (0.0974) | 1.5018 (0.3596) |  | 1.6709 (0.1330) | 1.7160 (0.1311) |  | 1.6309 (0.1659) | 1.5945 (0.3803) |
| %DET |  | 0.0335 (0.0087) | 0.0356 (0.0151) |  | 0.0330 (0.0043) | 0.0362 (0.0137) |  | 0.0322 (0.0077) | 0.0295 (0.0048) |  | 0.0317 (0.0042) | 0.0355 (0.0226) |

Notes: PRE: the pre-condition group; POST: the post-condition group.
