# Supplementary material for: Using nonlinear dynamics analysis to evaluate time response of cupping therapy with different intervention timings on reducing muscle fatigue
Source: Front Bioeng Biotechnol. 2024 Oct 1;12:1436235. doi: 10.3389/fbioe.2024.1436235 (PMC11473309; doi:10.3389/fbioe.2024.1436235)
Supplement: Supplementary file 3 [file DataSheet1.DOCX]

**Appendix A**

Specific inclusion and exclusion criteria for subjects were as follows. The inclusion criteria were participants aged 18–30 years had no painful restriction of upper limbs and participants did not experience the cupping therapy within the past three months. Criteria for exclusion were the general contraindications of cupping therapy, such as hemorrhagic disease, an open wound close to the cupping site, skin cancer, pregnant women, and the older individuals. Participants who had diagnosed cardiovascular, neurological (coronary insufficiency, arrhythmia, and heart failure), metabolic (diabetes mellitus), hypertension or musculoskeletal diseases. Participants were able to complete all visits and avoid other exercise training or other interventions for fatigue recovery throughout the study.
